# Audiological outcomes following middle cranial fossa repair of spontaneous cerebrospinal fluid leaks

**DOI:** 10.1017/S0022215124001294

**Published:** 2025-05

**Authors:** Jia Hui Ng, Arul Bala, Thomas Hendriks, Jafri Kuthubutheen

**Affiliations:** 1 Department of Otolaryngology and Head and Neck Surgery, Sir Charles Gairdner Hospital, Nedlands, Western Australia; 2 Department of Otolaryngology and Head and Neck Surgery, Singapore General Hospital, Singapore, Singapore; 3 Department of Neurosurgery, Sir Charles Gairdner Hospital, Nedlands, Western Australia; 4 Division of Surgery, University of Western Australia, Perth, Australia

**Keywords:** cerebrospinal fluid otorrhea, middle cranial fossa

## Abstract

**Objectives:**

The aim of this study is to investigate hearing outcomes in patients who have undergone cerebrospinal fluid (CSF) leak repair via a middle cranial fossa (MCF) approach and to identify any variables that influence post-operative hearing outcomes.

**Methods:**

This is a multi-centre study. A total of 65 patients who underwent an MCF approach CSF leak repair were included. Retrospective case review was conducted to collect patient demographic and clinical data including pre- and post-operative audiometry.

**Results:**

A total of 65 patients were included: 9 patients (9.2per cent) had an encephalocele confirmed on magnetic resonance (MR) imaging, whilst the remaining patients had biochemically confirmed, beta-trace protein positive CSF leaks. Post-operatively, there was a statistically significant improvement in both bone conduction (Z = -3.71, *p* < 0.001) and air conduction thresholds (Z = -5.82, *p* < 0.001). None of the studied variables were found to be associated with the degree of hearing improvement.

**Conclusion:**

The MCF approach for CSF leak repair yields favorable audiological outcomes.

## Introduction

Spontaneous cerebrospinal fluid (CSF) leaks of the temporal bone occur in the absence of any known prior trauma or surgery.^1^ This can be life threatening as it provides a portal of entry of microbes to enter the subarachnoid space, resulting in intracranial infection.^2^ Since earlier studies have shown that medical treatment such as prophylactic antibiotics are ineffective in preventing meningitis, early surgical intervention is favoured as the standard of care in most patients with CSF leak of the temporal bone.^3,4^

Numerous approaches have been proposed for the repair of CSF leak of the temporal bone. These include the middle cranial fossa (MCF) approach, the transmastoid approach, and the combined MCF-transmastoid approach. Previous studies on surgical outcomes have mainly focused on rates of CSF leak resolution, with only a handful of studies involving smaller patient numbers describing audiological outcomes. As unilateral conductive hearing loss is the most common presenting symptom of patients with spontaneous CSF leak of the temporal bone,^5^ audiological outcomes of CSF leak repair in this group of patients is of importance. This study aims to evaluate audiological outcomes in an MCF approach CSF leak repair.

## Methodology

This is a retrospective study conducted across 2 tertiary hospitals in Perth, Western Australia, between January 2016 and January 2022. A total of 65 adult patients who underwent MCF approach CSF leak repair for spontaneous CSF otorrhea in the study period were included. Pre-operatively, all patients had either had biochemically confirmed CSF leaks or an encephalocele confirmed on magnetic resonance imaging (MRI) imaging. Biochemical confirmation of CSF leak was obtained via aspiration of middle ear fluid and testing for beta-trace protein (BTP). All patients underwent pre-operative computed tomography (CT) imaging and most patients underwent pre-operative MRI imaging. Baseline pre-operative audiometry was also obtained.

All patients underwent the MCF approach CSF leak repair via a combined procedure between a neurosurgeon and a neurotologist, utilising a multilayer repair technique. Our technique is described in greater detail in a prior publication.^6^ Briefly, a linear supra-aural incision measuring approximately 6 to 9 cm with an endaural extension towards the external auditory meatus is used. The position of the craniotomy is marked with guidance from CT navigation by determining the location of the middle cranial fossa floor. A craniotomy measuring between 4 and 5 cm in the horizontal and vertical planes and centred along the external auditory meatus is made. Dura is then widely elevated over the middle fossa floor, with adequacy of dural elevation confirmed via CT navigation. Multi-layered repair is then undertaken with Duragen (Integra LifeSciences, Plainsboro, NJ) and Hydroset hydroxyapatite bone cement (Stryker, Kalamazoo, MI). Where necessary, TachoSil (Baxter, Deerfield, IL) was used to prevent contact of the bone cement to the ossicular chain. Post-operatively, a mastoid bandage was kept in situ for 48 hours, and the patients can be discharged after. All patients underwent regular surveillance in clinic by the treating clinicians. A post-operative audiogram was also obtained.

The medical records of the included patients were reviewed to obtain demographic data, examination findings, investigation results including pre- and post-operative audiometry, surgical outcomes and duration of post-operative follow up. Bone conduction (BC) and air conduction (AC) thresholds at 500, 1000, 2000 and 4000 Hz were used to compute BC and AC pure tone average (PTA) for each patient. The study was approved by the South Metropolitan Health Services Ethics Committee (approval number RGS0000001008). A waiver of consent was granted for the study in line with the NHMRC National Statement on Ethical Conduct in Human Research (2007).

Statistical analysis to determine any significant improvement in AC and BC hearing post-operatively was performed using the Wilcoxon signed rank test. The chi squared test and Mann–Whitney U test was used to determine if any variables were associated with audiological outcomes. SPSS version 23 (SPSS, Inc, an IBM Company, Chicago, Illinois) was used for all statistical analyses in this study.

## Results

A total of 75 patients underwent MCF approach repair of spontaneous CSF leak in the study period between January 2016 and January 2022; 10 patients were excluded due to incomplete data on pre-operative or post-operative audiometry. As such, 65 patients were included in this study. The median age of the patients was 65 years (interquartile range [IQR] 54.0–71.5). A total of 50.8 per cent of patients were female and 49.2 per cent were male. Median body mass index (BMI) was 26.9 (IQR 25.8–24.6). A total of 55.4 per cent of the surgical procedures were right sided, and the remainder were left sided. At presentation, 93.8 per cent of patients had fluid within the middle ear in the affected ear on examination, 90.8 per cent of patients were diagnosed with CSF otorrhea after positive testing for BTP on their middle ear aspirate, whilst the remaining 9.2 per cent of patients were diagnosed based on MRI finding of an encephalocele in the affected ear. All patients underwent a pre-operative CT scan and 92.3 per cent of patients underwent a pre-operative MRI scan. In all patients, the defect/s location was at the tegmen. This is summarised in Table 1.

**Table 1.** Patient demographics and clinical features

N=65	
Median age, in years (IQR)	65.0 (54.0–71.5)
Gender	
Male (%)	32 (49.2)
Female (%)	33 (50.8)
Median BMI (IQR)^a^	26.9 (25.8–24.6)
Side of CSF leak	
Right (%)	36 (55.4)
Left (%)	29 (44.6)
OME at presentation	
Yes (%)	61 (93.8)
No (%)	4 (6.2)
Pre-operative CT done	
Yes (%)	65 (100)
No (%)	0 (0)
Pre-operative MRI done	
Yes (%)	60 (92.3)
No (%)	5 (7.7)
Defect location in tegmen	
Yes (%)	65 (100)
No (%)	0 (0)
Indication for surgery	
BTP positive ME effusion (%)	59 (90.8)
Encephalocele on MRI (%)	6 (9.2)
^a^n=31, BMI information not available for 34 patients.	

Abbreviations: BMI = body mass index; BTP = beta-trace protein; CSF = cerebrospinal fluid; CT = computed tomography; IQR = interquartile range; MRI = magnetic resonance imaging; OME = otitis media with effusion.

Pre-operative median BC PTA was 22.5 dB (IQR 1.25–66.3) and median AC PTA was 41.3 dB (IQR 6.3–102.5). The median pre-operative air bone gap was 18.9 dB (IQR 9.4–26.4). Pre-operatively, 76.9 per cent of patients had any degree of hearing loss, and 73.9 per cent of patients had a significant degree of conductive hearing loss with an air-bone gap of 11 dB or more. This is summarised in Table 2.

**Table 2.** Pre- and post-operative audiometric data (N = 65)

**Pre-operative**	
Pre-operative PTA	
Median BC PTA (IQR)	22.5 dB (15.0–33.8)
Median AC PTA (IQR)	41.2 dB (28.8–55.0)
Median air-bone gap (IQR)	18.9 dB (9.35–26.4)
Pre-operative hearing (AC)	
Normal, 25 dB or better (%)	15 (23.1)
Mild, 26–40 dB (%)	17 (26.1)
Moderate, 41–60 dB (%)	21 (32.3)
Severe-profound, 61 dB or poorer (%)	12 (18.5)
Pre-operative air-bone gap	
10 dB or less (%)	17 (26.2)
11–25 dB (%)	31 (47.7)
26–50 dB (%)	17 (26.2)
**Post-operative**	
Post-operative PTA	
Median BC PTA (IQR)	18.6 dB (10.7–30.7)
Median AC PTA (IQR)	27.5 dB (17.5–42.5)
Median air-bone gap (IQR)	7.50 dB (5.00–12.4)
Post-operative hearing (AC)	
Normal, 25 dB or better (%)	28 (43.1)
Mild, 26–40 dB (%)	18 (27.7)
Moderate, 41–60 dB (%)	17 (26.2)
Severe-profound, 61 dB or poorer (%)	2 (2.08)
Post-operative air-bone gap	
10 dB or less (%)	45 (69.2)
11–25 dB (%)	18 (27.2)
26–50 dB (%)	2 (2.08)

Abbreviations: AC = air conduction; BC = bone conduction; IQR = interquartile range; PTA = pure tone average.

Post-operative audiometry was performed a median of 3.35 months (IQR 2.22–4.53) after surgery. The median post-operative BC PTA improved to 18.6 dB (IQR 1.0–66.0) from 22.5 dB (IQR 1.25–66.3) pre-operatively. Similarly, the median post-operative AC PTA improved to 27.5 dB (IQR 3.0–78.0), from 41.3 dB (IQR 6.3–102.5) pre-operatively. The audiological improvement was statistically significant for both BC PTA (Z = -3.71, *p* < 0.001) and AC PTA (Z = -5.82, *p* < 0.001). Post-operatively, the percentage of patients with any degree of hearing loss fell to 56.9 per cent, and only 29.3 per cent of patients still had a significant degree of conductive hearing loss with an air-bone gap of 11 dB or more. This is summarised in Table 2.

No difference in the degree of improvement of AC PTA was found between variables analysed, including male or female gender, presence or absence of pre-operative middle ear effusion, presence or absence of an encephalocele, and whether or not peri-operative ventilation tubes were used (*p* > 0.05).

No immediate failure of the repair was noted. However, delayed failure of the repair was noted in eight patients (12.3 per cent). The median time to failure was 14 months. There were no major post-operative complications. Minor complications included temporary facial nerve palsy that spontaneously recovered (n = 1) and temporary dysphasia secondary to temporal lobe edema (n = 1). The median length of follow-up was 13 months.

## Discussion

As unilateral conductive hearing loss is the most common presenting symptom of patients with spontaneous CSF leak of the temporal bone,^5^ hearing outcomes associated with the various described surgical approaches for CSF leak repair is of significance. Commonly described surgical approaches for CSF leak repair include the MCF approach, the transmastoid approach, and the combined approach. In our institution, the MCF approach is favoured for tegmen repair due to the ability to repair multiple defects and reinforce the integrity of the entire skull base. It also avoids violation of the middle ear cavity, removing the need for manipulation of the ossicular chain, which may cause conductive hearing loss in some cases.

This study is largest known to date describing audiological outcomes in patients undergoing the MCF approach repair of temporal bone spontaneous CSF leaks. In our series of 65 patients, there was a median PTA improvement and a median air-bone gap improvement of 11.3 dB and 8.70 dB, respectively, after surgery. This is congruent with two other similar studies published in the literature. Alwani *et al.* described his series of 24 patients (27 ears) who underwent the MCF approach for CSF leak repair and found a mean post-operative PTA improvement of 10.28 dB (*p* < 0.001),^7^ and a mean air-bone gap improvement of 9.31 dB (*p* < 0.001). Another similar study by Alwan *et al.* found that 10 patients demonstrated a mean improvement of 18.86 dB post-operatively.^8^ In addition, that study found that mean hearing improvement following CSF leak repair with fascial graft alone was 12 dB, which is poorer compared to 26.5 dB when multi-layered repair utilising fascial graft with bone was used.^8^ Our study is unable to draw conclusions on the impact of the technique of repair on hearing outcomes, as all patients in our study underwent a multi-layered repair technique.

Some authors have utilised a combined MCF/transmastoid approach for repair of CSF otorrhea. Stevens *et al.* described audiometric data in 25 patients and found that, although there was a statistically significant 6.1 dB improvement of post-operative air-bone gap (*p* = 0.05), post-operative mean PTA did not significantly improve.^9^ Another study by Son *et al.* described audiological outcomes in 19 patients who underwent combined a MCF/transmastoid approach repair. Only 15.8 per cent of patients had a more than 15 dB improvement of air-bone gap, and another 15.8 per cent had sensorineural hearing loss of more than 15 dB.^10^ Similarly, Yancey *et al.* reported audiometric outcomes in their series of 42 patients with spontaneous CSF otorrhea, with the majority (92.9 per cent) having undergone the combined MCF/transmastoid repair.^11^ No significant improvement in post-operative PTA was found. All of these studies describe significantly poorer hearing outcomes, in contrast to studies utilising an MCF-only approach for CSF leak repair. This could possibly be due to ossicular chain manipulation; in both studies, the transmastoid part of surgery involved division of the incudostapedial joint to facilitate dissection around the ossicles.

Only one study has directly compared audiological outcomes between an MCF approach for CSF leak repair to other approaches within their patient cohort. Ren *et al.* found that PTA improved by a mean of 15.6 dB in the 20 patients who underwent transmastoid approach repair, superior to an improvement of only 3.0 dB in the 8 patients who underwent an MCF approach repair (*p* = 0.01).^12^ However, this result may have been confounded, as 80 per cent of patients in that series undergoing transmastoid repair had posteriorly based skull base defects at the posterior fossa or tegmen mastoideum, and only 20 per cent had defects at the tegmen tympani. In contrast, 75 per cent of patients in the MCF group had defects in the tegmen tympani, closer to the native ossicular chain. In addition, impingement of the graft material on the ossicular chain during repair must be avoided to avoid compromising hearing outcomes.^10^

Our study found that variables like gender, presence or absence of pre-operative middle ear effusion, presence or absence of an encephalocele, and whether or not peri-operative grommet tubes were used did not impact hearing outcomes (*p* > 0.05). This is in concurrence with Yancey *et al.*, who similarly found that encephalocele involvement of and dissection from the ossicular chain did not appear to significantly influence hearing results.^11^ Ren *et al*. found that the aetiology of CSF leak (spontaneous vs. nonspontaneous), the type of skull base defect identified intra-operatively (single vs. multiple defects), and associated encephalocele (present vs. absent) did not seem to influence audiometric outcomes.^12^ In contrast, Stevens *et al*. concluded that patients with dehiscences in the posterior fossa have significantly greater improvements in PTA (*p* = 0.03) and air-bone gap (*p* = 0.05) post-operatively.^9^ This is similar to the conclusions drawn by Ren *et al*., who attributed improved audiologic outcomes in transmastoid repairs to the fact that the majority of such repairs were performed for posterior dehiscences.^12^

Interestingly, not only was a statistically significant improvement in AC PTA (Z = -5.82, *p* < 0.001) observed, a statistically significant improvement, albeit smaller, in BC PTA (Z = -3.71, *p* < 0.001) was also observed in this study. This is likely attributed to the Carhart's effect.^13^

Limitations of this study include its retrospective nature, variable timing of post-operative audiologic assessments, and the lack of long-term audiological data given the relatively short follow-up time. To overcome these limitations, future prospective studies can be undertaken with audiometric assessments performed at fixed intervals. Despite these limitations, this study remains the largest to date evaluation of audiologic outcomes in patients with spontaneous CSF otorrhea undergoing an MCF approach repair.

## Conclusion

The MCF approach for CSF leak repair allows the resurfacing of the entire tegmen and correction of tegmen defects of varying size, number, and location. While avoiding violation of the middle ear and disruption of the ossicular chain, it results in significant post-operative improvements in hearing.
